# Genotyping for Blast (*Pyricularia oryzae*) Resistance Genes in F_2_ Population of Supa Aromatic Rice (*Oryza sativa* L.)

**DOI:** 10.1155/2019/5246820

**Published:** 2019-11-15

**Authors:** L. Kanyange, J. Kamau, O. Ombori, A. Ndayiragije, M. Muthini

**Affiliations:** ^1^Department of Plant Sciences, Kenyatta University, P.O. Box 43844-00100, Nairobi, Kenya; ^2^International Rice Research Institute-Eastern and Southern Africa Office, P.O. Box 5132 Bujumbura, Burundi

## Abstract

The ascomycete fungus, *Pyricularia oryzae* or *Magnaporthe oryzae*, is known to cause blast disease in more than 80 host plants of the Gramineae family—cereals including rice and grasses. The improvement of the Supa234 rice line (IR97012-27-3-1-1-B, containing *badh2* gene for aroma) developed at IRRI-ESA Burundi consisted of introgression of R genes (*Pita* and *Pi9*) for blast resistance. The F_2_ population obtained via the cross had been screened for blast resistance using inoculation with *Pyricularia oryzae* spore's suspension. The objectives of this study were to assess the presence of *Pita* and *Pi9* genes for blast resistance and to assess the presence of the *badh2* gene for aroma in the screened F_2_ plants using molecular markers. Genotyping was carried out in 103 F_2_ plants which grew to maturity using the KASP genotyping method with SNP markers (snpOS0007, snpOS0006, and snpOS0022) targeting the *Pita* and *Pi9* genes for blast resistance and the *badh2* gene for aromatic fragrance. The genotyping results showed that 38 F_2_ plants had the *Pita* gene present in both alleles, 31 F_2_ plants with the *Pita* gene in one allele, and only one plant (3B1) was found with the *Pi9* gene in one allele. The *badh2* gene for aroma was detected in 27 F_2_ plants on both alleles and in 57 F_2_ plants on one allele. There were thirteen plants which had both the *Pita* gene and the *badh2* gene for aroma, and only one plant (3B1) had a combination of the three genes (*Pita*, *Pi9*, and *badh2*). Seven plants resistant to blast disease (2H2, 2H4, 1G2, 1C12, 1E13, 1B12, and 1C5) with the *Pita* and *badh2* genes were found, and only one resistant plant (3B1) had a combination of the three genes *Pi9*, *Pita*, and *badh2* which is recommended to be bulked for the development of the Supa aromatic rice variety resistant to blast disease. The plants generated by the best line 3B1 should further be evaluated for grain quality (Supa type) after F_5_ generation in the field.

## 1. Introduction

Rice (*Oryza sativa*) is a staple food worldwide for more than half of the world population [1]. Consumers prefer rice varieties with good grain quality like aroma, long grain, and amylase content. The aromatic trait enhances the market value of rice [2]. Nonaromatic rice has *badh2* gene in chromosome 8 encoding for betaine aldehyde dehydrogenase enzyme with 503 amino acids while in the aromatic one, the number of encoded amino acids is 251. The *badh2* gene produces *GABA*, a four-carbon nonprotein amino acid acting as a natural pesticide playing several roles including detoxification of free radicals, plant development, and plant defense [3]. The aromatic trait is coded by the mutant form of *badh2* gene with 8 bp deletion in exon 7 of the *badh2* gene, encoding a chemical compound 2-acetyl-1-pyrroline (2AP) [4–6]. The presence of the *badh2* mutant gene encoding for the pleasant aroma by producing 2-acetyl-1-pyrroline was established by Nadaf et al. [3] to be associated with some weakness like yield losses, sterility, and susceptibility to abiotic and biotic stresses including blast disease. The mutant form of the *badh2* gene has been associated with plant susceptibility to diseases as it suppresses the expression of the *badh2* gene [3]. Blast disease which is known to occur in 85 countries worldwide [7, 8] is manifested in temperate and humid regions as the main cause of reduction of rice production [9, 10]. The blast disease can cause high yield loss of 10 to 85% when factors or enhancers of epidemic development (high mean temperature, relative humidity higher than 85-89%, the presence of dew, excessive nitrogen fertilization, and drought stress) are present [11].

Methods used in controlling blast disease include adjustment of planting time, burning diseased tissues, use of healthy seeds, and cultural systems like fungicide and fertilizer management without ignoring the use of resistant plant varieties bearing genes for blast resistance [12, 13]. Molecular screening of major rice blast resistance genes has been determined using molecular markers, which showed close-set linkage to 11 major rice blast resistance genes (*Pi-d2*, *Pi-z*, *Piz-t*, *Pi-9*, *Pi-36*, *Pi-37*, *Pi5*, *Pi-b*, *Pik-p*, *Pik-h*, and *Pi-ta2*), in a collection of 32 accessions resistant to *Magnaporthe oryzae* [13]. Out of the 32 accessions, the *Pi-d2* and *Pi-z* appeared to be omnipresent and gave positive expression. The analysis of QTLs links genetic markers with DNA base variations, like single-nucleotide polymorphisms (SNPs) and simple sequence repeats (SSRs) or microsatellite to the QTLs of interest [14]. The most popular markers used in QTL analysis are SSRs, also known as microsatellites. However, SSR markers have been replaced by SNPs as molecular markers of choice in plant genetic analysis due to their codominant inheritance, their biallelic nature, chromosome-specific location, and genome-wide distribution [15]. The objective of this study was to assess the presence of R genes (*Pita* and *Pi9*) for blast resistance and the *badh2* gene for aroma in the F_2_ generation using rice genotyping methods. In this study, markers (SNP and InDels) linked to the *Pita* and *Pi9* genes conferring blast resistance and the *badh2* gene for aroma inherited in the improved parent (Supa aromatic 234) were genotyped using the KASP method in 103 F_2_ plants which had been phenotypically screened for blast resistance.

## 2. Materials and Methods

### 2.1. Rice Seeds

In this study, seeds of 208 individual rice plants of the F_2_ population and 72 seeds of parent or control lines (12 of Supa234, 12 of Vuninzara, 12 of Gigante, 12 of IRBL9-W, 12 of CO39, and 12 of BC3) were obtained from IRRI-ESA Burundi. The F_2_ generations had been developed by IRRI-ESA breeders in Burundi for the purpose of improvement of Supa234 rice (IR97012-27-3-1-1-B) aromatic line for resistance to blast disease. The IRBL9-W, a highly resistant parental monogenic line with the *Pi9* gene, and BC3 bearing the *Pita* gene were used as resistant controls. The Supa234 and CO39 rice lines without genes for blast resistance were used as susceptible controls. Supa234 line (IR97012-27-3-1-1-B, aromatic) containing the *badh2* gene for aroma was also used as a positive control for aromatic fragrance. The plants were grown in a randomized complete block design (RCBD) in trays. Rice seeds were sown in Minuro trays (plastic trays 36 cm wide and a depth of 56 cm) filled with soil collected from Gihanga rice-growing areas. The soil was dried under the sun for two weeks, to diminish plant contamination, and then ground. Each Minuro tray had 104 wells (each with a size < 40 cm^3^) arranged in 8 rows and 13 columns. The seeds were sown at a rate of four per well which were later thinned to one after germination. One hundred and thirty-five plants selected from 208 F_2_ plants, four parents (Supa234, Vuninzara, Gigante, and IRBL9-W), and two controls (CO39 and BC3) were screened for blast resistance at vegetative and reproductive stages. The screening was carried out into petri dishes by inoculating detached leaves with blast spores using the spot inoculation method [16]. Five microliters of conidial/spore suspension were inoculated on both sides of each leaf segment. Each plant sample had its own negative control, which were inoculated with a mixture of Tween 20 and deionized water. After inoculation, the petri dishes containing the leaf segments were maintained at 25 ± 1°C under continuous fluorescent light (11 to 12 *μ*Em^−2^ s^-1)^ for 24 hours. Excess moisture on the leaflets was removed after 24 h by blotting with sterile pieces of laboratory tissue paper [16]. The leaves were then incubated at 25 ± 1°C in the dark room for 10 days. To maintain the moisture level, sterile distilled water was added once every 3 days to the petri dishes to avoid desiccation of the leaf segments during incubation.

### 2.2. DNA Isolation

Leaf samples from the 135 selected F_2_ individual plants, parents, and controls were collected 21 days after planting. The leaf samples were lyophilized to remove moisture and kept at -80°C. The DNA for genotyping was extracted from the leaf disks of 103 F_2_ plants (which grew to maturity) among the 135 plants screened for blast resistance, parents, and controls. Parents and the control lines CO39 and BC3 were also screened. IRBL9-W, Vuninzara, and Gigante parents and the BC3 rice line were used as positive controls for the *Pi9* and *Pita* gene markers while Supa234 parents were used as the positive control for the *badh2* gene maker. BC3 and Supa234 were also used as negative controls. Leaf tissues weighing 0.5 g from each sample were used to extract genomic DNA. Leaf samples were separately crushed using a mortar and pestle, and the powdered samples were collected in sterilized 1.5 ml Eppendorf tubes. In each tube, 400 *μ*l of CTAB lysis buffer containing 6.25 mM of potassium ethyl xanthogenate, 0.5% CTAB, 700 mM NaCl, 10 mM EDTA, and 100 mM Tris, pH 7.5, was added and mixed by vortexing for 30 s. The tubes were then incubated for 1 hour in a water bath at 65°C. An equal volume of chilled chloroform isoamyl in the ratio of 24 : 1 was added to each tube and centrifuged for 10 min at 13,000 rpm at 4°C. The supernatant was then transferred to new sterilized Eppendorf tubes. In each tube, 400 *μ*l of isopropanol was added and kept overnight at -20°C for nucleic acid precipitation. This was followed by centrifugation at 13,000 rpm at 4°C for 8 minutes. The liquid phase was then gently decanted off leaving the DNA pellets. The DNA pellets were washed by adding 400 *μ*l of 70% ethanol followed by centrifugation at 13,000 rpm. The 70% ethanol was then decanted, and the samples air-dried by inverting the Eppendorf tubes on sterilized laboratory tissues. Finally, DNA was dissolved using 100 *μ*l of TE buffer containing 10 mM Tris, pH 7.5, and 0.5 mM EDTA. The extracted DNA was stored at -21°C before genotyping. The quality and quantity of DNA was determined using agarose gel electrophoresis using 0.8% agarose.

### 2.3. Genotyping

The extracted genomic DNA samples were genotyped using the Kompetitive Allele Specific PCR (KASP) genotyping technique [17] in the Intertek laboratory in Sweden. The KASP markers for the *Pi9* and *badh2* genes used were designed by IRRI (Table 1). The SNP-specific KASP assay mix, the universal Master mix (genotyping mixture), and the DNA sample used for all PCR reactions had a total volume of 10 *μ*l. In 96-well plates for the PCR, one well contained a mixture of 5 *μ*l genotyping mixture (4.4 *μ*l of 2x KASP Master mix and 0.6 *μ*l of KASP assay mix) and 5 *μ*l of 50 ng DNA from each sample. Each KASP assay mix comprised three assay-specific nonlabelled oligonucleotides specific to a SNP or InDel marker comprising two allele-specific forward primers and one common reverse primer. Each primer harbored a unique tail sequence corresponding with a universal fluorescence resonant energy transfer (FRET) cassette and a primer-tail was labelled with FAM dye while on the tail of the second primer was labelled with HEX dye. The KASP Master mix on the other hand contained two universal FRET cassettes (HEX and FAM), ROX (passive reference dye), free nucleotides, Taq polymerase, and MgCl_2_ in an optimized buffer solution.

The Kompetitive Allele Specific PCR genotyping was performed in the following conditions according to Devran et al. [18]: one cycle for hot activation at 94°C for 15 min and the DNA denaturation was performed in 10 cycles at 94°C for 20 sec. The primer annealing and elongation were performed in 10 cycles for 60 seconds by dropping the temperature from 61 to 55°C at a rate of 0.6°C per each cycle. After, the temperature was raised to 94°C for 20 secs in 26 cycles to allow new denaturation and then lowered to 55°C for 60 seconds during annealing and elongation. When the amplification reactions were completed, 5 *μ*l of the amplified products was transferred into the 384-well plates and detected on a BMG PHERA Star plate reader with a fluorescent resonance energy transfer (FRET) using the genotype cluster analysis Kraken caller software from LGC Genomics assigning a genotype to each produced color. Geotypes were scored according to the guideline of Table 2.

### 2.4. Data Analysis

The traits associated with each genotype and the positions of each SNP marker were generated by R software [19]. Based on the genotypic traits, a numerical scoring method was used assigning 1 to a positive allele and 0 for a negative allele. The scores were used to calculate the genotypic relationship between the parents and the F_2_ populations and analysis of molecular variance (AMOVA) using GenAlex software version 6.5 [20]. Principal coordinate analysis (PCoA) showing the genetic differentiation between the rice plants was generated using GenAlex software version 6.5. A dendrogram showing the relationship between the plants was drawn based on the genetic dissimilarity using the neighbor-joining method using Darwin software version 6 [21].

## 3. Results

### 3.1. Molecular Marker Results

The genotyping results of the rice plants are presented in Table 3. There were 39 plants with the *Pita* gene for blast resistance represented on two alleles (score 1 : 1); among them, 23 plants including 1C2, 1G2, 1C5, 1H7, 1D5, and 1B12 were either resistant or highly resistant in both stages of development (vegetative and reproductive) (Table 3). The *Pita* gene was also present in allele 1 in 30 plants; among them, 10 were resistant or highly resistant in both stages including 3B1, 1D1, 3E5, and 1D8 (Table 3). The IRBL9-W parent had the *Pi9* gene for blast resistance in both alleles (score 1 : 1) while only one F_2_ plant, 3B1, had the *Pi9* gene in only one allele (score 1 : 0) (Table 3). The Supa234 (IR97012-27-3-1-1-B, the aromatic), Gigante parents, and 27 F_2_ plants had the *badh2* gene for aroma present in both alleles (score 1 : 1); among them, 14 plants including 1G2, 1C5, 3E5, and 1B12 were resistant or highly resistant in both stages. There were fifty-seven F_2_ plants including 1A1, 3B1, 1F11, and 1A6 which had the *badh2* gene present in one allele (score 1 : 0) (Table 3). There were also plants including 1C1, 3C6, and 3E8 that did not have any of the targeted genes *Pita*, *Pi9*, and *badh2* (Table 3).

Among the resistant or highly resistant F_2_ plants, only one plant, 3B1, had a combination of three genes (*Pita*, *Pi9*, and *badh2*), each present in one allele and nine plants (1G2, 1C5, 1D5, 1B12, 1C12, 1E13, 2B2, 2H2, and 2H4) having a combination of *Pita* and *badh2* genes in both alleles (Table 3). However, there were 4 plants, 1H3, 2H5, 1G11, and 4C6, which did not possess the targeted R genes (*Pita* and *Pi9*) for blast resistance but were resistant to blast disease in both stages.

### 3.2. Genetic Variation between Plants

For the analysis of genetic variation within the screened plants, GenAlEX Software version 6.5 [20] grouped the categories into populations where F_2_ plants were grouped in population 1, Gigante parent in population 2, Vuninzara in population 3, Supa234 (IR97012-27-3-1-1-B) in population 4, and IRBL9-W in population 5. The BC3 control plants were grouped in population 6 and CO39 in population 7. Based on the genotype scores, the genetic variation calculated between the populations indicated that the number of observed alleles per locus (Na) ranged between 0.00 and 2.00 while the number of effective alleles (Ne) per locus ranged from 1 to 1.49 (Table 4). The F_2_ plants (pop 1) had the highest number of effective alleles (Ne) (1.49) while all the other rice populations only had one (1) effective allele.

The F_2_ population had the maximal percentage of polymorphic loci (% P) (100%) while the parents and controls had no polymorphic loci (% P) (0%) (Table 4). In this study, high genetic diversity was observed in F_2_ rice plants (pop 1) with the mean Shannon's Information Index *I* = 0.41 while within the parents, there was no genetic diversity (*I* = 0) (Table 4). The mean expected heterozygosity (He) ranged from 0 for populations 2, 3, 4, 5, 6, and 7 (parents and control's lines) to 0.28 for pop 1 (F_2_ plants) (Table 4).

### 3.3. Analysis of Molecular Variance (AMOVA)

The analysis of molecular variance (AMOVA) for the seven populations showed that the genetic variation among populations (52%) was slightly higher compared to that within populations (48%). However, the variations were not significant (*P* > 0.05) (Table 5).

### 3.4. Principal Coordinate Analysis

**Figure 1:**
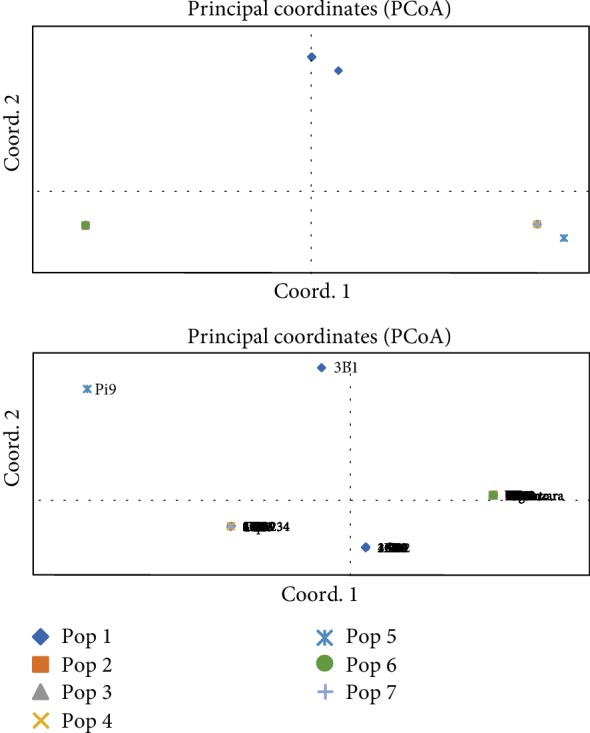
Principal coordinate analysis (PCoA) for the 103 F_2_ rice plants, 23 parent plants, and 19 control plants. Percentage variation explained by the three axes, 1: 83.71%; 2: 11.59%; 3: 4.70%.

The principal coordinate analysis (PCoA) of 103 F_2_ plants, 23 plants from 6 parents, and 19 plants from 2 controls populations clustered differently in the PCoA. The IRBL9-W parent (pop 5) with the *Pi9* gene was in its own cluster but in the same quadrant I with the F_2_ plant 3B1 (Figure 1). However, the parents Vuninzara (pop 3) and Gigante (pop 2) bearing the *Pita* gene clustered together with the BC3 control (pop 6) in quadrant II while Supa234 (IR97012-27-3-1-1, aromatic) parent (pop 4) clustered with CO39 control (pop 7) without any R gene in quadrant IV. Other F_2_ plants clustered with Supa234 parent and CO39 and the remaining part of F_2_ population clustered alone in the PCoA (quadrant III) (Figure 1). There was no genetic differentiation between F_2_ plant 3B1 and IRBL9-W (*Pi9*) plants. There was also no genetic differentiation between Supa234 parent (IR97012-27-3-1-1) and CO39 control and some of the F_2_ plants (Figure 1). There was no genetic differentiation between parents Vuninzara and Gigante and the control BC3 as they clustered in the same quadrat (Figure 1). The F_2_ plants were distributed in three clusters (quadrants I, III, and IV) while the other populations were found in only one quadrat (Figure 1).

### 3.5. Phylogenetic Analysis

**Figure 2:**
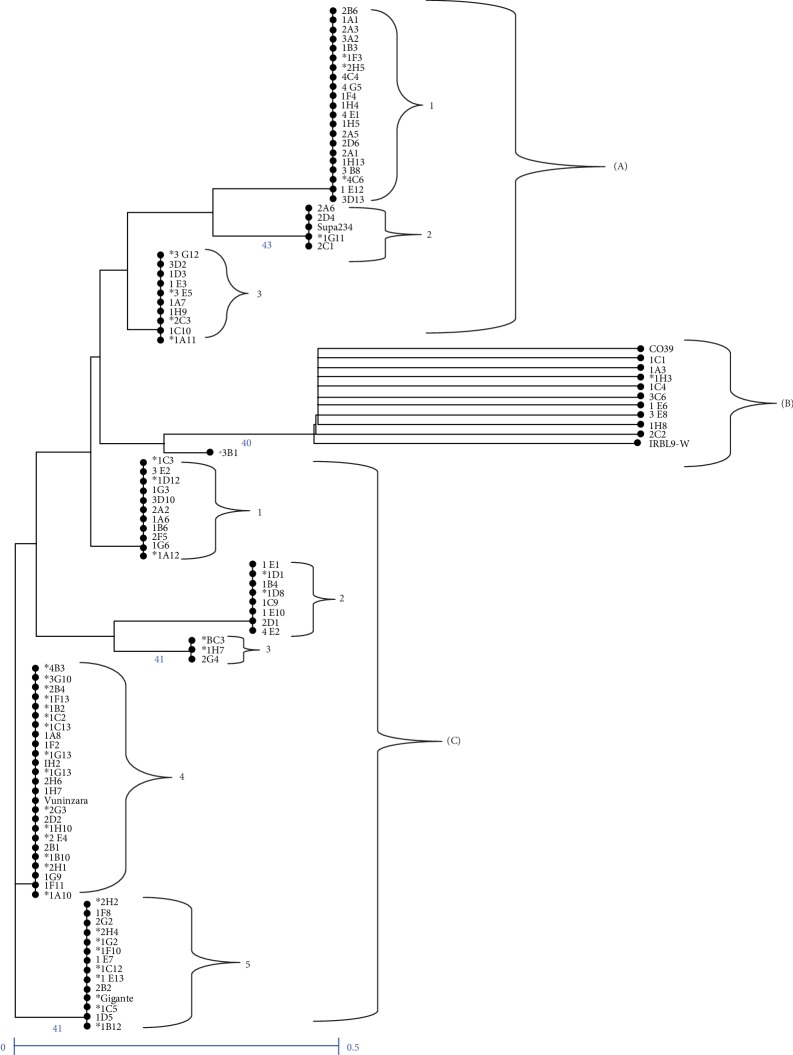
Dendrogram for the 103 F_2_ rice plants, parents (Supa234, Gigante, Vuninzara, and IRBL9-W) and controls (CO39 and BC3). Letters: cluster; numbers: subclusters. ∗: resistant or highly resistant plants. The number shown at the node of the dendrogram indicates the percentage of bootstrap support from 1000 iterations. Bootstrap values above 40% are the only ones that are shown.

The neighbor-joining phylogenetic tree based on the genetic dissimilarity grouped the 103 F_2_ plants into three main clusters (clusters A, B, and C) (Figure 2). Cluster A containing Supa234 parents with the *badh2* gene for aroma consisted of 35 F_2_ plants including the resistant plants like 1A11, 2C3, 3G12, 3E5, 4C6, and 1G11. Cluster A had three subclusters (1, 2, and 3) in which subcluster 2 contained Supa234 clustering with 4 F_2_ plants with bootstrap support of 43% (Figure 2). The smallest cluster B contained the negative control CO39, IRBL9-W (*Pi9* gene donor parent) both without the *Pita* gene nor the *badh2* gene and 9 F_2_ plants which are supported by 40% bootstrap except plant 3B1 (Figure 2). The third cluster C composed of mainly parents and controls with the *Pita* gene (BC3 control in subcluster 3, Vuninzara parent in subcluster 4, and Gigante parent in subcluster 5) and 58 F_2_ plants. The cluster C had 5 subclusters in which subcluster 3 supported by 41% bootstrap contained BC3 which was the positive control with the *Pita* gene.

The subcluster 4 contained the Vuninzara parent containing the *Pita* gene for blast resistance and subcluster 5 supported by 41% bootstrap value contained Gigante (*Pita* gene donor parent). Twenty resistant F_2_ plants clustered together with Vuninzara and Gigante parents in subclusters 4 and 5. In subcluster 4 containing Vuninzara which has the *Pita* gene, clustered resistant 13 plants including 1A10, 2H1, 1B10, 2E4, 1H10, 2G3, 1B13, 1G13, 1C2, 1F13, 2B4, 4B3, and 3G10. Subcluster 5, in which Gigante is clustered (parent with *Pita* gene), contained 7 blast resistant plants including 2H2, 2H4, 1G2, 1C12, 1E13, 1B12, and 1C5 (Figure 2).

## 4. Discussions

Marker-assisted selection is a breeding technique in which selection is done base on genotype of a marker of dominant or recessive alleles within a population [22]. In this study, marker-assisted selection was used in order to identify F_2_ plants of Supa aromatic rice line which may contain *Pita* and *Pi9* genes for blast resistance and *badh2* gene for aroma. KASP genotyping showed that *Pita* gene was predominant in the F_2_ population (except one plant 3B1 with *Pi9* gene) even though not all plants had *Pita* gene and not all the positive plants for *Pita* gene were homozygous in both alleles (Table 3). The homozygous genotypes (resistant: resistant or resistant/homozygous) contained the *Pita* gene represented in both alleles and heterozygous (resistant: susceptible or resistant heterozygous) genotypes were characterized by the presence of the *Pita* gene on one allele while in the homozygous genotypes (susceptible: susceptible) *Pita* gene was absent in both alleles. This shows the state of segregation within the F_2_ population. This finding concurs with those reported by Jia et al. [23], in which there was a segregation in the F_2_ population for the *Pita* gene (resistant/heterozygous and resistant/homozygous). The *badh2* gene for aroma was detected in 84 F_2_ plants in both allele or on one allele which demonstrate the inheritance of aroma from parent and segregation. In the present study, there were plants which had both the aroma and blast resistance genes, similar to the findings by Luo et al. [24] who reported a successful development of WH6725 resistant line to blast disease which possessed both genes. In the present study, the presence of *Pita* gene in resistant or moderately resistant plants may be attributed to the fact that *Pita* gene has been found to confer a medium-spectrum resistance [25].

The analysis carried out on genetic diversity and gene frequencies in the seven populations of plants used in this study (F_2_ plants, Gigante plants, Vuninzara plants, Supa234 plants, IRBL9-W plants, CO39 plants, and BC3 plants) showed genetic diversity (Shannon's Information Index, *I* = 0.410) within population 1 (F_2_ plants) while in parent's populations, the Shannon's Information Index *I* was zero (Table 4). That genetic diversity ranging from 0 to 0.410 show a moderate diversity in the screened plants compared to the moderate genetic diversity ranging from 0.05 to 0.78 observed in fifty SSR markers used in germplasm of fifty red rice by Islam et al. [26]. The heterozygosis of zero found in parents and controls lines demonstrated that parents used in the cross carried out at IRRI-ESA were 100% homozygous or true breeding [27]. However, heterozygosis of 0.28 observed in F_2_ plants is associated to the state of segregation of F_2_ generation (called segregating population by Mendel).

Analysis of molecular variance (AMOVA) in the rice plant populations showed that there was a slightly higher variation among populations (52%) although variation was also observed within populations (48%) however the variations were not significant (*P* > 0.05). The low genetic variation among the population and within population, respectively, indicate that the plants under this study were closely related. This variation found is different from the reports on variations among population (34%) and within population (66%) [28], and among groups (35.28%) and within groups (64.72%) reported by Chakhonkaen et al. [29].

The F_2_ plants clustered in dendrogram and in PCoA according to the presence of R genes and aroma in alleles. According to the principal coordinate analysis (PCoA), there were no genetic differentiations between 3B1 F_2_ plant and IRBL9-W plants gene due to the presence of *Pi9* gene in the 3B1 and IRBL9-W plants. This was also observed in the dendrogram where the two plants, 3B1 and IRBL9-W clustered in the vicinity. However, they were separated due to the presence of a single copy of *Pita* and *badh2* in 3B1. The fact that Supa234 (IR70212-27-3-1-1-B) parent and CO39 control clustered together is due to the absence of the target R genes; hence, the F_2_ plants clustering together do not contain the R genes. The F_2_ plants were distributed in three plot areas as they were distributed in three cluster of dendrogram due to the fact their genetic characteristics differ where some offspring carried genes from one parent while others had genes from both parents and others did not have any of the targeted three genes (*Pita*, *Pi9*, and *badh2*).

## 5. Conclusion and Recommendation

The molecular marker genotyping of the rice plants for R genes for blast resistance shows the presence of *Pita* gene conferring resistance to blast disease in many F_2_ plants, represented in either both alleles or on one allele. The *Pi9* gene was recovered in only one F_2_ plant 3B1 (represented on one allele). Resistant rice plants including F_2_ population of Supa234 (IR702-23-3-1-1-B, aromatic line) bearing *badh2* gene for aroma and *Pita* gene for blast resistance were identified in this study. The resistant F_2_ line 3B1 obtained in this study combining *Pi9*, *Pita*, and *badh2* genes can be used for development of an aromatic rice variety (Supa type) with resistance to blast disease.

Based on the above findings, there is a need to for further study on the resistant 3B1 F_2_ plant identified with the 3 targeted genes (*Pita*, *Pi9*, and *badh2*), by testing for blast resistance in field conditions to assess the stability of the resistance. The resistant 3B1 plant, found with aroma gene and resistance genes can be studied further for grain quality (Supa type) in final stage of variety fixation. Further research is necessary to check for other R genes which can be the source of resistance in the five resistant plants with *badh2* gene for aroma (1G11, 3E5, 1A11, 3G12, and 2C3) which did not contain the targeted R genes (*Pita* and *Pi9*) or contained a single copy of the *Pita* gene.

## Figures and Tables

**Table 1:** The primer sequences for SNP and InDel markers used in genotyping [19].

Gene	Marker ID	Primer name	Primer sequence	Favourable allele	Unfavourable allele
*Pi9*	snpOS0007	InDel-F	CGCCGGTTGATAAGTAAAAGCT TGATTATGTTTTTTATGTGGGG	—	CGATGGTTTC
		InDel-R	CAAGAACTAATATCTACCCATGG		
*Pita*	snpOS0006	SNP-F	CCGTGGCTTCTATCTTTACCTG CCGTGGCTTCTATCTTTACCTT	C	A
		SNP-R	AGTCAGGTTGAAGATGCATAGA		
*badh2*	snpOS0022	InDel-F	ACATAGTGACTGGATTAGGTTCTG CTGGTAAAAAGATTATGGCTTCA	TATAT	AAAAGATTATGGC
		InDel-R	CATCAACATCATCAAACACCACT		

SNP: single-nucleotide polymorphism; F: forward primer; R: reverse primer; InDel: insertion/deletion.

**Table 2:** Characteristics associated with the SNP/InDel markers.

SNP/InDels	Genes	Chr	Position	Markers	Allele 1	Allele 2	Genotype 1	Genotype 2	Trait of allele 1	Trait of allele 2	Score of allele 1	Score of allele 2
**Pi-ta**	*Pita*	12	10607554	snpOS0006	A	C	A:A	C:C	Susceptible	Resistant	0	1
**Pi9-1b**	*Pi9*	6	10381489	snpOS0007b	CGATGGTTTC	—	CGATGGTTTC:CGATGGTTTC	-:-	Susceptible	Resistant	0	1
**BADH2.1-7**	*badh2*	8	20382865	snpOS0022	AAAAGATTATGGC	TATAT	AAAAGATTATGGC:AAAAGATTATGGC	TATAT:TATAT	Not fragrant	Fragrant	0	1

Key: SNP: single-nucleotide polymorphism; InDel: insertion/deletion; Chr: chromosome. Alternative alleles existing in the target loci and corresponding genotype.

**Table 3:** Determination of the presence or absence of *Pita* and *Pi9* genes for blast resistance and *badh2* gene for aroma in F2 rice plants.

	Marker gene					
	snpOS0006		snpOS0007b		snpOS0022	
	*Pita*		*Pi9*		*badh2*	
Plant	AL 1	AL 2	AL 1	AL 2	AL 1	AL 2
**1A1**	0	0	0	0	1	0
^∗^ **3B1**	1	0	1	0	1	0
**1C1**	0	0	0	0	0	0
^∗^ **1D1**	1	0	0	0	0	0
**1 E1**	1	0	0	0	0	0
**3A2**	0	0	0	0	1	0
**1B2**	1	1	0	0	1	0
^∗^ **1C2**	1	1	0	0	1	0
**3D2**	1	0	0	0	1	1
**3 E2**	1	0	0	0	1	0
**1F2**	1	1	0	0	1	0
^∗^ **1G2**	1	1	0	0	1	1
**1H2**	1	1	0	0	1	0
**1A3**	0	0	0	0	0	0
**1B3**	0	0	0	0	1	0
^∗^ **1C3**	1	0	0	0	1	0
**1D3**	1	0	0	0	1	1
**1 E3**	1	0	0	0	1	1
**1F3**	0	0	0	0	1	0
**1G3**	1	0	0	0	1	0
^∗^ **1H3**	0	0	0	0	0	0
**1B4**	1	0	0	0	0	0
**1C4**	0	0	0	0	0	0
**1F4**	0	0	0	0	1	0
**1H4**	0	0	0	0	1	0
^∗^ **1C5**	1	1	0	0	1	1
^∗^ **1D5**	1	1	0	0	1	1
^∗^ **3 E5**	1	0	0	0	1	1
**1H5**	0	0	0	0	1	0
**1A6**	1	0	0	0	1	0
**1B6**	1	0	0	0	1	0
**3C6**	0	0	0	0	0	0
**1 E6**	0	0	0	0	0	0
**1G6**	1	0	0	0	1	0
**1A7**	1	0	0	0	1	1
**1D7**	1	1	0	0	1	0
**1 E7**	1	1	0	0	1	1
^∗^ **1H7**	1	1	0	0	0	0
**1A8**	1	1	0	0	1	0
**3 B8**	0	0	0	0	1	0
^∗^ **1D8**	1	0	0	0	0	0
**3 E8**	0	0	0	0	0	0
**1F8**	1	1	0	0	1	1
**1H8**	0	0	0	0	0	0
**1C9**	1	0	0	0	0	0
**1G9**	1	1	0	0	1	0
**1H9**	1	0	0	0	1	1
^∗^ **1A10**	1	1	0	0	1	0
^∗^ **1B10**	1	1	0	0	1	0
**1C10**	1	0	0	0	1	1
**3D10**	1	0	0	0	1	0
**1 E10**	1	0	0	0	0	0
**1F10**	1	1	0	0	1	1
^∗^ **3G10**	1	1	0	0	1	0
^∗^ **1H10**	1	1	0	0	1	0
^∗^ **1A11**	1	0	0	0	1	1
**1F11**	1	1	0	0	1	0
^∗^ **1G11**	0	0	0	0	1	1
^∗^ **1A12**	1	0	0	0	1	0
^∗^ **1B12**	1	1	0	0	1	1
^∗^ **1C12**	1	1	0	0	1	1
^∗^ **1D12**	1	0	0	0	1	0
**1 E12**	0	0	0	0	1	0
^∗^ **3G12**	1	0	0	0	1	1
^∗^ **1B13**	1	1	0	0	1	0
**1C13**	1	1	0	0	1	0
**3D13**	0	0	0	0	1	0
^∗^ **1 E13**	1	1	0	0	1	1
^∗^ **1F13**	1	1	0	0	1	0
^∗^ **1G13**	1	1	0	0	1	0
**1H13**	0	0	0	0	1	0
**2A1**	0	0	0	0	1	0
**2B1**	1	1	0	0	1	0
**2C1**	0	0	0	0	1	1
**2D1**	1	0	0	0	0	0
**4 E1**	0	0	0	0	1	0
^∗^ **2H1**	1	1	0	0	1	0
**2A2**	1	0	0	0	1	0
^∗^ **2B2**	1	1	0	0	1	1
**2C2**	0	0	0	0	0	0
**2D2**	1	1	0	0	1	0
**4 E2**	1	0	0	0	0	0
**2G2**	1	1	0	0	1	1
^∗^ **2H2**	1	1	0	0	1	1
**2A3**	0	0	0	0	1	0
^∗^ **4B3**	1	1	0	0	1	0
^∗^ **2C3**	1	0	0	0	1	1
^∗^ **2G3**	1	1	0	0	1	0
^∗^2B4	1	1	0	0	1	0
4C4	0	0	0	0	1	0
2D4	0	0	0	0	1	1
^∗^2 E4	1	1	0	0	1	0
2G4	1	1	0	0	0	0
^∗^ **2H4**	1	1	0	0	1	1
**2A5**	0	0	0	0	1	0
**2F5**	1	0	0	0	1	0
**4G5**	0	0	0	0	1	0
^∗^ **2H5**	0	0	0	0	1	0
**2A6**	0	0	0	0	1	1
**2B6**	0	0	0	0	1	0
^∗^ **4 C6**	0	0	0	0	1	0
**2D6**	0	0	0	0	1	0
**2H6**	1	1	0	0	1	0
^∗^ **Gigante**	1	1	0	0	1	1
**Vuninzara**	1	1	0	0	1	0
**Supa234**	0	0	0	0	1	1
^∗^ **IRBL9-W**	0	0	1	1	0	0
^∗^ **BC3**	1	1	0	0	0	0
**CO39**	0	0	0	0	0	0

The plants with ^∗^: 37 F_2_ plants; resistant parent and control plants which were either resistant or highly resistant in both stages; others are moderately resistant and moderately susceptible. 1A1 to 2H6: F_2_ plants; Gigante and Vuninzara: parent donor of *Pita* gene; Supa234: recipient parent with *badh2* gene for aroma; IRBL9-W: *Pi9* gene donor parent; BC3: positive control for *Pita* gene; CO39: negative control for all target genes (*Pita*, *Pi9*, and *badh2*) genes. AL 1: allele 1; AL 2: allele 2.

**Table 4:** Means of different allele (Na), number of effective alleles (Ne), Shannon's Information Index (*I*), expected heterozygosity (He), unbiased expected heterozygosity (UHe), and percentage of polymorphic loci (% P) of the rice plant populations (F_2_ rice plants, Gigante, Vuninzara, Supa234, IRBL9-W donor, BC3, and CO39).

Pop	*N*	Na	Ne	*I*	He	uHe	% P
Pop 1	103	2.00 ± 0.00	1.49 ± 0.27	0.410 ± 0.20	0.28 ± 0.14	0.28 ± 0.14	100
Pop 2	6	0.67 ± 0.33	1.00 ± 0.00	0.00 ± 0.00	0.00 ± 0.00	0.00 ± 0.00	0
Pop 3	6	0.67 ± 0.33	1.00 ± 0.00	0.00 ± 0.00	0.00 ± 0.00	0.00 ± 0.00	0
Pop 4	5	0.00 ± 0.00	1.00 ± 0.00	0.00 ± 0.00	0.00 ± 0.00	0.00 ± 0.00	0
Pop 5	6	0.33 ± 0.33	1.00 ± 0.00	0.00 ± 0.00	0.00 ± 0.00	0.00 ± 0.00	0
Pop 6	7	0.67 ± 0.33	1.00 ± 0.00	0.00 ± 0.00	0.00 ± 0.00	0.00 ± 0.00	0
Pop 7	12	0.00 ± 0.00	1.00 ± 0.00	0.00 ± 0.00	0.00 ± 0.00	0.00 ± 0.00	0

Key: *N*: no. of plants per population; Na: no. different alleles; Ne = 1/(*p*^2^ + *q*^2^); *I* = −1∗(*p*∗Ln(*p*) + *q*∗Ln(*q*)); He = 2∗*p*∗*q*; UHe = (2*N*/(2*N* − 1))∗He. Pop 1: composed of 103 F_2_ rice plants genotyped; pop 2: represent Gigante parent containing *Pita* gene; pop 3: Vuninzara parent containing *Pita* gene; pop 4: Supa234 (aromatic) recurrent parent blast susceptible; pop 5: IRBL9-W, a *Pi9* gene donor parent; pop 6: BC3, a positive control for *Pita* gene; pop 7: CO39, a negative control for all genes.

**Table 5:** Analysis of molecular variance (AMOVA) for the 145 rice plants of the seven categories: 103 F_2_ plants, 23 parent plants, and 19 control plants based on genotyping genomic DNA.

Source	Df	MS	Est. var.	% mol var.	*P* value
Among pops	6	4.676	0.373	52%	<0.5175
Within pops	138	0.348	0.348	48%	<0.4372
Total	144		0.721	100%	

Df: degree of freedom; MS: mean square; est. var.: estimated variance; pops: populations; % mol var.: percentage molecular variance.

## Data Availability

The data used to support the findings of this study are available from the corresponding authors upon request.
